# Nomophobia, Psychopathology, and Smartphone-Inferred Behaviors in Youth With Depression: Longitudinal Study

**DOI:** 10.2196/57512

**Published:** 2025-02-19

**Authors:** Tianyi Zhang, Andres Camargo, Lianne Schmaal, Vassilis Kostakos, Simon D'Alfonso

**Affiliations:** 1 School of Computing and Information Systems University of Melbourne Melbourne Australia; 2 Centre for Youth Mental Health University of Melbourne Melbourne Australia

**Keywords:** mobile sensing, nomophobia, digital phenotyping, depression, mental health, smartphone use, personal sensing, behavior analysis, machine learning, mobile health, mobile phone

## Abstract

**Background:**

Smartphones have become an indispensable part of people’s lives, and the fear of being without them, what has been termed “no mobile phone phobia” (nomophobia), is a growing phenomenon. The rise of problematic smartphone use highlights the urgent need to explore the intricate relationship between smartphones and human behavior. However, the connections between nomophobia, mental health indicators, smartphone use patterns, and daily activities remain largely underexplored.

**Objective:**

This study aimed to explore the relationship between young adults with depression and smartphones and investigate nomophobia by analyzing data obtained from a pilot study of depression in a youth cohort. Exploring nomophobia can enhance our understanding of the dynamics between young adults and smartphone use, potentially empowering them to manage and regulate their smartphone use more effectively.

**Methods:**

During an 8-week period, data collected via smartphone sensors, such as locations and screen status, were gathered from a cohort of 41 individuals diagnosed with major depressive disorder. In addition to passive-sensing smartphone data, the study collected ecological momentary assessments and psychometric measures, including the Nomophobia Questionnaire, which formed the basis of our investigation. We explored statistical associations among smartphone-derived behavioral features, psychometric indicators, and nomophobia. In addition, we used behavioral and psychometric data to develop regression models demonstrating the prediction of nomophobia levels.

**Results:**

Our findings revealed that the level of nomophobia was positively associated with depression and negative affect, lower geolocation movements, and higher comfort with smartphone sensing. The exploratory predictive linear regression models demonstrated the feasibility of predicting an individual’s Nomophobia Questionnaire score based on their smartphone sensing data. These models effectively used input features derived from both a combination of smartphone sensing data and psychometric measures and from smartphone sensing data alone.

**Conclusions:**

Our work is the first to explore the relationship between nomophobia and smartphone sensor data. It provides valuable insights into the predictors of nomophobia level, contributing to the understanding of the relationship between smartphones and human behavior and paving the way for future studies.

## Introduction

### Background

*Nomophobia* is a portmanteau of “no mobile phone phobia” and describes the fear of being without a smartphone. The concept of nomophobia has been proposed and researched in several early works [1,2], and a subsequent paper [3] presented a validated quantitative scale to measure it, known as the Nomophobia Questionnaire (NMP-Q). The rapid proliferation of smartphones has made nomophobia a timely phenomenon to investigate.

By July 2022, more than two-thirds of the world’s population used mobile phones [4]. However, the popularity of smartphones has raised concerns about their impact on individuals’ well-being. Smartphone addiction, especially among youth populations, has become a prominent issue. Approximately 95% of teenagers (aged 13-18 years) use cell phones every day in the United States [5]. It is claimed that overattachment to smartphones can lead to actual harmful outcomes such as depression [6], anxiety [7], and reduction in physical activity in the young population.

The notions of problematic smartphone use and smartphone addiction remain unsettled and need further exploration to determine their status as genuine diagnostic phenomena. The last decade has seen various attempts to quantify these notions using self-report scales [8]; however, more work is needed to better understand and predict nomophobia. One promising avenue is the analysis of nomophobia in terms of *digital footprints*, a term used to denote the data trails generated from human interactions with digital devices (eg, smartphones) and the internet (eg, social media). The idea of computationally mining digital footprints to infer an individual’s (or a group’s) behavior, preferences, and mental states has given rise to a field of research associated with the terms *digital phenotyping* [9], *personal sensing* [10], and *psychoinformatics* [11].

In this study, we investigated the relationship among nomophobia, smartphone sensor data, several mental health measures, and several debriefing questions concerning participant comfort with personal data sensing. This investigation was based on the collection of passive smartphone sensing data, ecological momentary assessment (EMA) responses, and various psychometric measures collected at baseline and the 8-week follow-up of a mental health digital phenotyping study of 41 young people diagnosed with major depressive disorder (MDD). We studied this information in relation to accompanying results on the NMP-Q.

### Related Work

Research suggests that nomophobia has been a prevalent phenomenon across youth populations. According to previous investigations, up to 99% of students or young people have some degree of nomophobia [12-16], and a quarter of university students experience severe nomophobia [17,18]. This, in turn, adversely affects their mental or physical health and performance [19], even to the extent that there have been proposals to include nomophobia in the *Diagnostic and Statistical Manual of Mental Disorders, Fifth Edition* [20].

As a nascent field, investigation into nomophobia is still emerging [19,21]. One study [22] used structural equation modeling and semantic network analysis to investigate the cognitive factors associated with nomophobia, showing that people with higher nomophobia tend to “perceive smartphones as their extended selves” [22] due to personal memories. Examining the relationships between nomophobia and psychological assessments, researchers have found various associations between nomophobia and social media use [23], loneliness [23-25], smartphone addiction [26], stress [25], problematic phone use [27], personality traits (Big Five personality traits) [28] and emotional stability [28], depression [29], anxiety [24,29], hyperactivity [29], social phobia [30], and oppositional symptoms [29]. One study [31] found that vertical collectivism, which involves sacrificing personal goals and submitting to authorities, is positively associated with nomophobia. Another study [32] took a qualitative approach and found a strong correlation between smartphone addiction and personal feelings such as social anxiety, antisocial behaviors, and loneliness.

In terms of nomophobia measurement, the basis of our work is the NMP-Q scale [3]. This validated scale consists of 4 dimensions: communication, connection, informativeness, and convenience. The 140-point total on the scale represents the sum of the responses to 20 questions, each rated on a Likert scale from 1 to 7. As per the scale developers, the total score can be categorized into 4 nomophobia severity levels: none and mild, moderate, and severe nomophobia. Since its release, this scale has been used in a handful of studies that affirm the reliance on mobile phones as an emerging issue for young people [16,32-34].

A recent study [35] investigated the NMP-Q scale in terms of various independent variables, particularly certain psychopathological measures, smartphone use, and lifestyle measurements. The study identified interpersonal sensitivity, obsession-compulsion, and the number of hours of smartphone use per day as predictors of nomophobia. While these results are of interest, the study was limited in terms of smartphone sensing. Only a few smartphone use–related inputs were obtained, such as the number of years using a smartphone, the number of daily hours using a smartphone, and the most used apps. Furthermore, the method to measure smartphone use was based on simply asking participants to subjectively provide estimates of these figures. While these results are of interest, the investigation of nomophobia against a greater variety of objectively collected smartphone sensing data is called for.

Thus, building upon this earlier work, in this study, we explored nomophobia outcomes against a range of objectively collected smartphone data inputs and psychometric scale results. Unlike work in digital phenotyping that uses smartphone data to infer psychopathology, our research on nomophobia uses objective phone data to directly characterize patterns of smartphone use.

## Methods

### Study Design

The data for this study were obtained from the SMARTSENSE-D project [36]. Participants were recruited from primary and specialized youth mental health services in Melbourne, Victoria, Australia, over a 2-year period between 2020 and 2021. Recruitment took longer than anticipated due to the COVID-19 pandemic situation in Melbourne. In total, 41 young people (aged 16-25 years) with a diagnosis of MDD consented to participate in data collection for 8 weeks.

With consent from their clinicians, we contacted potential participants who met the diagnosis criteria for MDD based on results from the Mini-International Neuropsychiatric Interview [37] and Patient Health Questionnaire–9 [38]. We included participants with an MDD diagnosis based on the *Diagnostic and Statistical Manual of Mental Disorders, Fifth Edition* [39], who regularly used a smartphone, understood smartphone data tracking, and were willing to participate. We excluded those at high risk of suicide, actively manic or actively psychotic, not familiar enough with smartphones to complete daily EMA surveys, or with insufficient ability to communicate in English.

Each participant provided demographic information, psychometric assessments (before and after the 8-week period), continuous smartphone sensing data (Android phones only), and data from a wrist actigraphy device during the 8-week trial period. We included in our investigation score results from the following measures and questionnaires that were administered in the study: (1) a demographic questionnaire (baseline assessment only) that captured demographic information such as age, gender, educational level, and other relevant background information; (2) the Depression, Anxiety, and Stress Scale–21 (DASS-21) [40,41], a set of 3 self-reported scales designed to measure the emotional states of depression, anxiety, and stress; (3) the Quick Inventory of Depressive Symptomatology (QIDS) [42,43], a brief self-report tool that assesses the severity of depressive symptoms; (4) the University of California, Los Angeles (UCLA), Loneliness Scale [44,45], which is designed to measure subjective feelings of loneliness and social isolation; (5) the Penn State Worry Questionnaire (PSWQ) [46,47], a self-report measure that assesses the tendency to worry excessively; (6) the Rumination Response Scale (RRS) [48,49], which assesses the extent to which individuals focus on their depressive symptoms and the possible causes and consequences of these symptoms; (7) the NMP-Q [3,50], which measures the severity of nomophobia, which is the fear of being without a mobile phone; and (8) a debriefing questionnaire (follow-up assessment only), which used a Likert scale from 1 (*strongly disagree*) to 7 (*strongly agree*) to capture participant attitudes toward such smartphone sensing and their perspectives on its feasibility and acceptability and also captured participant attitudes regarding each of the sensors being activated on their phones using a Likert scale from 1 (*extremely uncomfortable*) to 7 (*extremely comfortable*).

The AWARE-Light smartphone sensing app [51] was installed on each participant’s Android phone and continuously collected geolocation, keyboard, communication (through calls and messages), screen status, app use, and touch operation data using sensors [52,53]. In addition, the AWARE-Light app administered EMA surveys twice daily at noon and 8 PM. The debriefing questionnaire items and EMA questions can be found in Multimedia Appendices 1 and 2, respectively.

### Ethical Considerations

This study involved the collection of primary data directly from participants. All participants provided informed consent, which included detailed information about the purpose of the study, the types of data collected, how the data would be stored securely, and how they would be used. Participants were informed that their data would remain confidential and anonymized, and they had the option to withdraw from the study at any time without consequence. The study protocol was reviewed and approved by the University of Melbourne Human Research Ethics Committee (ID: 1955691.4). This study was carried out in accordance with the principles contained in the Declaration of Helsinki, the Australian National Health and Medical Research Council National Statement on Ethical Conduct in Human Research, and the National Health and Medical Research Council Australian Code for the Responsible Conduct of Research.

Participants were reimbursed Aus $100 (US $62.32) upon completion of the study, as well as an additional Aus $1.5 (US $0.93) for completion of each EMA survey (maximum of Aus $126 [US $78.53] if all surveys were completed).

### Data Processing

#### Preprocessing

As participants installed the AWARE-Light sensing app at different times on their first study day, we discarded the data collected during the initial day of the 56-day data collection period. Therefore, the analysis was based on 55 full 24-hour days for each participant starting from the second day of installation.

We handled missing values in 2 steps. First, we only retained the sensor data for a participant if the data were recorded for at least 27 out of the 55 days; if a participant’s sensor captured data for ≤26 days, then the data were excluded. The phone call and SMS text message sensors were exempt from this requirement as they may not be used every day. As such, we had different numbers of participants with valid data for each sensor dataset. Given this filtering, we ended up focusing on the smartphone sensor data of participants whose location and screen sensors met the threshold, resulting in a total of 27 valid sets of participant data.

Second, we used imputation to handle missing values in active data, including EMA questions and psychological assessments. In sum, <2% of the psychometric data were missing and imputed, and approximately 30% of EMA data were missing values. The days with missing EMA answers were excluded from feature calculations, such as the average and SD.

To resolve the missing data in the QIDS, we left the missing field as null to represent the missing data. Because the questions in these scales vary significantly, we believe that participants’ attitude toward certain questions cannot be represented by the ratings for other questions in the same scale. Note that the total value of the QIDS remained unchanged after resolving the missing data.

To resolve the missing data in assessments other than the QIDS, we first calculated the average ratings of nonmissing questions for each participant and then used the average score to replace the missing data. In this way, we assumed that participants had no special preference toward the questions they skipped.

For psychometric assessments, missing data arose from participants selecting “Prefer not to say,” which we treated as missing data. This suggests that the missingness might be related to the participants’ unwillingness to disclose certain information, implying that the data were missing not at random. Given that <2% of the data were missing, we opted for a straightforward imputation method. While this approach does not explicitly account for the potential missing-not-at-random nature of the data, the small proportion of missing data minimized the impact on our overall analysis. Nevertheless, we acknowledge that more sophisticated methods such as pattern-mixture models or sensitivity analysis could be considered in future studies to address any potential biases.

We verified the correlation and root mean square error (RMSE) values for the psychometric data and found that the lowest correlation between the original and imputed datasets was 0.89, whereas the highest correlation corresponded to an RMSE of 0, indicating a near-perfect match. Specifically, the RMSE values for the baseline DASS-21 total, baseline UCLA Loneliness Scale total, baseline PSWQ total, baseline RRS total, and follow-up RRS total were 0.61, 0.94, 2.15, 0.78, and 2.56, respectively. This indicates that the overall patterns remained robust and intact after imputation.

#### Feature Generation

The smartphone sensor data collected were used to generate a variety of information features. Message, keyboard, and screen status features were generated using the RAPIDS feature generation tool (AWARE Framework) [54], and the library by Doryab et al [55] was used to generate geolocation features such as average speed, maximum length staying at clusters, and number of frequent places [56]. Specifically, the Hierarchical Density-Based Spatial Clustering of Applications With Noise (HDBSCAN) [57] algorithm was used for cluster generation, effectively handling noise and clusters of varying densities.

We first generated daily smartphone sensor data and then averaged the data across the study period. For instance, to calculate the “maximum time spent at significant locations” feature, we first determined the maximum length of time that each participant stayed in various clusters per day, and then we computed the average duration over the 56 days for each participant.

In addition, we created our own Python scripts to work with certain sensors or generate features that were not available using the aforementioned tools, including touch operations, screen status, app use, and daily EMA questions. The fundamental features are the counts and average values of the interaction with the phone, such as counts of clicks and scrolling from touch operations, average and SD of EMA values, and the number of screen unlocks during each hour of the day.

The features derived from passively collected smartphone sensor data were preselected based on their relevance and potential to provide meaningful insights into user behavior and nomophobia. Location-based features offer detailed insights into the daily movement and habits of participants, encompassing various aspects of mobility, such as variability, speed, distance, and location diversity. Message traces and keyboard use for information retrieval or exchange indicate the extent of users’ reliance on their smartphones. Screen use time and frequency, count of touch actions, and app use for particular app types not only reflect smartphone engagement but also serve as potential indicators of problematic smartphone use [27].

Regarding the psychometric measures investigated (DASS-21, QIDS, UCLA Loneliness Scale, PSWQ, RRS, and NMP-Q), apart from using the aggregate score of an assessment scale, in certain cases, we also investigated the subscores based on the dimensions or domains in each scale. For example, the four dimensions in the nomophobia scale [27] are (1) not being able to communicate (belonging and connectedness), (2) losing connectedness, (3) not being able to access information, and (4) giving up convenience. Similarly, several of the other psychometric assessments, such as the DASS-21, QIDS, and RRS (see the Models section), were also composed of different dimensions.

It is worth noting that we focused on analyzing the psychometric measures from the follow-up assessment for several reasons. First, the follow-up assessment results aligned better with the phone sensor data in terms of chronological order. The follow-up assessments were chronologically recorded after the passive sensing and could reveal more information about the entire study period. Second, the follow-up assessments from various questionnaires aligned better with each other as they were taken at the same time point. Third, the psychometric measures changed little throughout the 56-day study duration. The ratio of the absolute scale difference (calculated using the formula [after − before]/before) for the DASS-21, PSWQ, RRS, and QIDS was approximately 0.1, whereas the ratio for the UCLA Loneliness Scale was <0.05. This subtle difference between the baseline and follow-up absolute values indicates only minor variations across these scales, suggesting stability in the measured psychometric attributes over the study period.

Apart from the total scores for each psychometric measure, we explored the subfactors because they offered a more detailed and sensitive view of the data. While the total score provides a holistic perspective, the subfactors reveal detailed insights into different aspects of the psychometric constructs. These subfactors can highlight specific areas of change that may not be apparent in the overall scores, offering a more granular understanding. Therefore, we were interested in examining the correlation and predictive power of both the total scores and the subfactor scores of the psychometric measures.

#### Models

We used the Spearman rank correlation [58] to investigate the statistical associations between nomophobia scores and other variables of interest. The Spearman correlation was chosen due to the nonnormal distribution of ordinal Likert-type questionnaire measures and continuous smartphone-collected features. We adjusted all *P* values using the original Benjamini-Hochberg method [59] to control the false discovery rate at 20%. This adjustment allows up to 20% of the rejected null hypotheses to be false positives and was chosen to ensure a reasonable balance between sensitivity and specificity in our exploratory analysis.

To test the correlations between smartphone sensor features and nomophobia, we only included a participant’s dataset for a sensor if it had at least 27 out of the 55 days of availability (location and screen sensors were selected after this filtering process as they were the 2 sensors with sufficient data). Correlations between psychometric and debriefing questionnaire features and the NMP-Q were explored using valid data for 41 participants. Correlations between EMA features and nomophobia were explored using valid data for 85% (35/41) of the participants.

In total, we selected 48 correlation pairs to investigate, as shown in Textbox 1. The significant feature pairs are presented in the Results section.

We generated the smartphone sensor and psychometric features (Multimedia Appendix 3) shown in Textbox 2.

**Selected pairs of features for correlation investigation.**

- A total of 9 location sensor features were each paired with the Nomophobia Questionnaire (NMP-Q) total score (Multimedia Appendix 3).
- A total of 3 screen sensor features were each paired with the NMP-Q total score (Multimedia Appendix 3).
- A total of 8 ecological momentary assessment (EMA) features were each paired with the NMP-Q total score. Each feature was obtained by averaging the morning and evening scores of an EMA affect question (Multimedia Appendix 3).
- A total of 2 debriefing questionnaire features—one based on the question concerning overall comfort with using AWARE-Light (statement: “I felt comfortable using the app”) and the other based on the average of questions concerning comfort with the accelerometer, app, communication, location, light, keyboard, network, and screen sensors—were paired with the NMP-Q total score as well as the NMP-Q subdimension scores for a total combination of 10 pairs (Multimedia Appendix 3).
- A total of 4 Depression, Anxiety, and Stress Scale–21 (DASS-21) features were each paired with the NMP-Q total score (the DASS-21 total score, as well as the scores for the 3 subdimensions of depression, stress, and anxiety). Each subdimension or factor was added to the exploration given that they each represented separate mental health conditions. Furthermore, this investigation had a particular focus on depression given the cohort of young adults with depression (Multimedia Appendix 3).
- A total of 11 Quick Inventory of Depressive Symptomatology (QIDS) features were each paired with the NMP-Q total score (the QIDS total score, as well as the scores for the 10 subdimensions). As with the DASS-21, given the focus on depression, it was decided to investigate the subdimensions of this scale as well (Multimedia Appendix 3).
- One Rumination Response Scale total score feature was paired with the NMP-Q total score (Multimedia Appendix 3).
- One Penn State Worry Questionnaire total score feature was paired with the NMP-Q total score (Multimedia Appendix 3).
- One University of California, Los Angeles, Loneliness Scale total score feature was paired with the NMP-Q total (Multimedia Appendix 3).

**Smartphone sensor and psychometric features generated.**

**Location**

- The RAPIDS tool (AWARE Framework) used Density-Based Spatial Clustering of Applications With Noise algorithms (HDBSCAN) to calculate location points into clusters. On the basis of the time spent in each location cluster, 8 features derived from location clusters were explored in correlation with the Nomophobia Questionnaire (NMP-Q) scale. In total, 5 of these features describe significant places (the minimum, maximum, average, and SD of the time spent per day at significant locations), as well as the number of visited significant places per day. The other 3 features include the average speed (indicating how fast a person moves) per day, total traveled distance per day, and time spent at home per day. There may be some overlap of information if the home is considered the most significant location for the person. The maximum period of staying at significant locations refers to the longest duration that a user stays at any identified significant location over a given period, identifying key locations that are important to the user, such as their home, workplace, or frequently visited places. Extended stays at certain locations can provide insights into routines and lifestyle patterns. The SD of the time spent in a cluster reflects the degree of variability in the time participants spend at different location clusters, with a higher SD indicating greater variation.

**Screen**

- A screen use episode is defined as the period from when the phone is unlocked until the screen is turned off. A total of 3 screen features were explored for correlation: the total duration of unlock episodes, considered as the time when smartphones are actively used; the longest duration of any unlock episode, representing the longest use episode per day; and the number of all unlock episodes, indicating how many times the person unlocked their smartphone in a day. For predictive models, 3 additional features were considered: the minimum, average, and SD of unlock episode duration in a day.

**Messaging**

- In total, 3 pairs of messaging features were explored for predictive models, including the total number of messages received from or sent to the contact who sent messages most frequently per day, the total number of messages received and sent by the participant per day, and the number of different contacts from whom the participant received and sent messages per day.

**App use**

- A total of 4 app use features were included in the candidate pool for the predictive model: the total number of times that social media apps were used by the participant per day, the total number of times that communication apps (eg, messaging and email) were used per day, the total number of times that entertainment apps (eg, games and videos) were used per day, and the total number of times that music or audio apps were used per day.

**Touch actions**

- One feature derived from collected smartphone touch data was the average number of items scrolled. This metric is calculated by tracking the number of distinct scroll actions a user performs on their smartphone screen during a specified period.

**Psychometric subfactors**

- Our study included both the total scores and subfactor scores of the following psychometrics: Depression, Anxiety, and Stress Scale–21 (DASS-21); Quick Inventory of Depressive Symptomatology (QIDS); University of California, Los Angeles (UCLA), Loneliness Scale; Penn State Worry Questionnaire (PSWQ); Rumination Response Scale (RRS); and NMP-Q. However, not all combinations across the subfactors were explored. We primarily focused on the total score on the NMP-Q for exploring correlations with smartphone sensor features and other psychometrics. In addition, for specific debriefing questions, we examined subfactors of participants’ NMP-Q scores to understand why they felt comfortable with sensitive data being collected via smartphones. The DASS-21 is designed to be interpreted using 3 subscales: depression, anxiety, and stress. The QIDS also includes subfactors even though some subfactors are represented by a single question. In contrast, the RRS, PSWQ, and UCLA Loneliness Scale do not have subfactor representations and, thus, were not explored in this context.

In general, we investigated the correlation between each of these features and the NMP-Q total score. The one exception to this was the debriefing questionnaire features, which we also tested against the NMP-Q subdimensions because of our preassumption that using AWARE-Light and sensor activation might naturally correlate with certain aspects of nomophobia, such as the need for communication or convenience.

Apart from the correlation analysis, we also experimented with constructing some supervised machine learning models with the follow-up nomophobia scores as the outcome to be predicted. In our supervised learning models, we only included data from participants whose location and screen sensor data met the threshold of at least 27 out of the 55 days of availability (which resulted in 27/41, 66% of the participants included in the analysis). If a participant met this requirement for both sensors, then data for all their sensors, their psychometric results, and their EMA data were retained for regression.

After data cleaning, linear regression was applied to 47 extracted features (Multimedia Appendix 4) from 66% (27/41) of the participants, with filtering based on the combination of location and screen sensors. We focused on exploring two general models: (1) those whose input was only passive smartphone sensor features and (2) those whose input combined passive and active data, including EMA, phone sensors, and psychometrics. We implemented both wrapper methods and filter methods to test various combinations of features using *R*^2^ to evaluate the models. Of the 47 preselected features, we included those that showed a potential improvement in model performance of >0.01 in terms of *R*^2^ results. To predict NMP-Q scores, we used leave-one-out cross-validation for model evaluation due to the small sample size. In addition to the *R*^2^ score, we used mean absolute error (MAE), mean absolute percentage error (MAPE), and RMSE to assess the regression errors.

## Results

### Data Description

**Figure 1:**
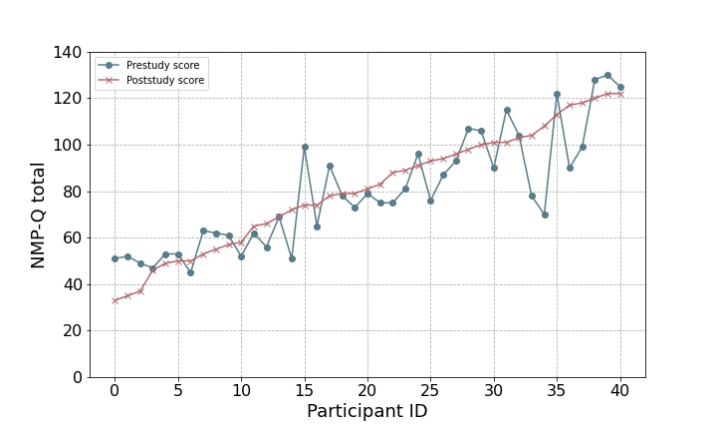
Comparison of Nomophobia Questionnaire (NMP-Q) total scores among 41 participants with major depressive disorder at baseline and at the end of the 8-week longitudinal study period, revealing minimal differences.

In total, 41 individuals took part in the study. Their demographic characteristics are presented in Table 1. The means and SDs of the psychometrics are shown in Table 2. The NMP-Q total scores of the participants only slightly changed between baseline and follow-up, as shown in Figure 1, with a Spearman test-retest reliability of 0.87 and a Cronbach α of 0.93. A total of 4 nomophobia severity levels as defined in the study by Yildirim and Correia [3] were used: no nomophobia (score of 0-20), mild nomophobia (score of 21-59), moderate nomophobia (score of 60-99), and severe nomophobia (score of 100-140). All participants (41/41, 100%) had an NMP-Q score of >20, with most participants (18/41, 44%) reporting a moderate level of nomophobia. A similar percentage of participants reported a mild (11/41, 27%) or severe (12/41, 29%) level of nomophobia.

**Table 1:** Demographic information of the young adults with depression involved in the 8-week longitudinal study (N=41).

Characteristic and categories			Participants, n (%)
**Sex at birth**			
	Female	29 (71)	
	Male	12 (29)	
**Age group (y)**			
	16-18	11 (27)	
	19-22	17 (41)	
	23-25	13 (32)	
**Country of birth**			
	Australia	35 (85)	
	Overseas (South America, Asia, Europe, and New Zealand)	6 (15)	

**Table 2:** Means, SDs, and score ranges of the psychometric measurements taken at the end of the 8-week study among 41 participants for the Depression, Anxiety, and Stress Scale–21 (DASS-21); Penn State Worry Questionnaire (PSWQ); Rumination Response Scale (RRS); University of California, Los Angeles (UCLA), Loneliness Scale; and Quick Inventory of Depressive Symptomatology (QIDS).

Psychometric scale	Scores, mean (SD; range)
DASS-21	30.6 (12.9; 0-126)
PSWQ	59.1 (12.9; 16-80)
RRS	26.9 (5.5; 4-40)
UCLA Loneliness Scale	57.0 (5.0; 20-80)
QIDS	15.0 (5.5; 0-27)

### Statistical Analysis

Exploratory data analysis was conducted using the Spearman rank correlation analyzing follow-up NMP-Q results against the psychometric, questionnaire, passive smartphone sensing, and EMA data. As detailed previously, we examined 48 feature pairs, with each pair involving the NMP-Q total score or that of one of its subfactors. Of these 48 tested combinations, we found 11 (23%) significant results that are presented and analyzed in this section, with the remaining 37 (77%) tests yielding nonsignificant results (Multimedia Appendix 3).

To qualify for inclusion as significant, the results needed to have *P*≤.05 and a correlation coefficient of >0.3 or <−0.3, in line with the standard by Cohen [60], according to which results of >0.3 are of moderate effect.

Overall, significant correlations with the NMP-Q were found for certain EMA items, certain geolocation features, certain debriefing questions, and certain psychometric scores. The following is a descriptive summary of these correlation results.

Regarding location data (Table 3), there was a positive correlation between nomophobia (NMP-Q total score) and time spent at home, time spent at the most significant location, the maximum time spent in a cluster (at a daily level, this feature captures the maximum time that a participant spent at any of their significant locations [as determined by the geolocation clustering algorithm] for the day), and the SD of the time spent in a cluster (significant location; at a daily level, this feature captures the SD of all the durations of time that a participant spent at their significant locations for the day).

Regarding debriefing questions (Table 4), a positive correlation was found between the comfort level of a participant using the sensing app and their nomophobia in terms of the NMP-Q factor of giving up convenience, and a positive correlation was found between the comfort level of a participant having certain sensors tracking their personal information and their nomophobia in terms of the NMP-Q factor of giving up convenience.

Regarding the DASS-21 (Table 5), there was a positive correlation between the depression score and nomophobia (NMP-Q total score).

Regarding the QIDS (Table 5), mood and fatigue in the depressive cohort were positively correlated with nomophobia (NMP-Q total score).

Regarding EMA (Table 6), participants who felt more relaxed and happier daily reported lower nomophobia (NMP-Q total score).

**Table 3:** Significant correlations between Nomophobia Questionnaire total scores and smartphone-based location sensor features among 27 participants with major depressive disorder during the 8-week study period.

Location feature	*P* value	Spearman correlation coefficient
The maximum period staying at significant locations	.02	0.35
The SD of the time spent in a cluster	.04	0.32
Time spent at home	.02	0.35
Time spent at the most significant location	.03	0.35

**Table 4:** Significant correlations between the “giving up convenience” subfactor of the Nomophobia Questionnaire and participants’ responses to debriefing questions at the conclusion of the 8-week study period observed among 41 participants with major depressive disorder.

Feature	*P* value	Spearman correlation coefficient
Participant comfort with using AWARE-Light (item 4 in Multimedia Appendix 1)	.02	0.37
Participant comfort with sensor activation (an average of items 21-28 in Multimedia Appendix 1)	.01	0.40

**Table 5:** Significant correlations between Nomophobia Questionnaire total scores and various psychometric measures assessed among 41 participants with major depressive disorder at the conclusion of the 8-week study period.

Psychometric measure	*P* value	Spearman correlation coefficient
DASS-21^a^ factor—depression	.02	0.38
QIDS^b^ factor—mood	.046	0.31
QIDS factor—energy and fatigue	.02	0.37

^a^DASS-21: Depression, Anxiety, and Stress Scale–21.

^b^QIDS: Quick Inventory of Depressive Symptomatology.

**Table 6:** Significant correlations between Nomophobia Questionnaire total scores and responses to ecological momentary assessment (EMA) questions captured over the 8-week study period among 41 participants with major depressive disorder.

EMA measure	*P* value	Spearman correlation coefficient
Average of morning and evening EMA score for how happy participants felt	.03	–0.34
Average of morning and evening EMA score for how relaxed participants felt	.03	–0.337

### Predicting Nomophobia

**Figure 2:**
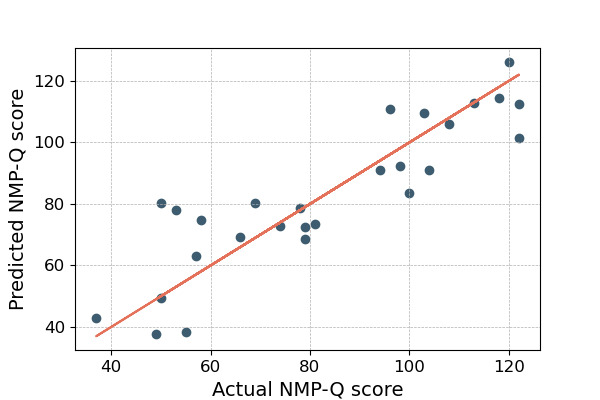
Comparison of actual versus predicted Nomophobia Questionnaire (NMP-Q) total scores using a combination of psychometric measures and smartphone sensor data from 27 participants with depressive symptoms who had valid data during the 8-week study period. This highlights the potential of machine learning for accurate predictions.

**Figure 3:**
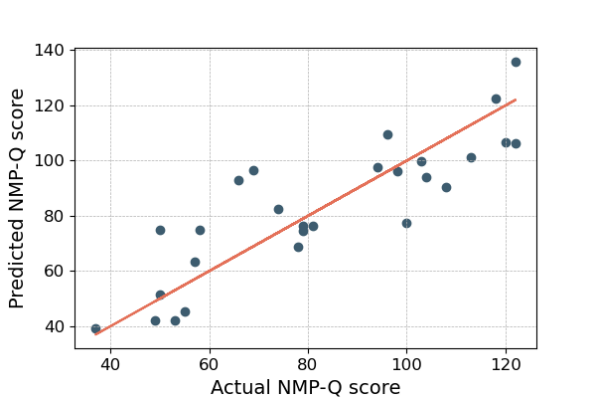
Comparison of actual versus predicted Nomophobia Questionnaire (NMP-Q) total scores using only smartphone sensor data from 27 participants with depressive symptoms who had valid data during the 8-week study period without incorporating psychometric measures. This demonstrates the feasibility of machine learning–based predictions.

Tables 7 and 8 show the attributes of our best models for passively plus actively collected data and passively collected data only, respectively. Our best model achieved an *R*^2^ value of 0.779 (MAE=9.42; MAPE=13.56%; RMSE=12.09). Figure 2 shows the distribution of predicted and actual NMP-Q scores based on both phone sensor features and psychometric measures input into a linear regression model. We also developed a model to predict individuals’ NMP-Q scores using only smartphone sensor data. Our best model achieved an *R*^2^ of 0.732 (MAE=10.88; MAPE=14.44%; RMSE=13.32), as illustrated in Figure 3.

**Table 7:** Overview of smartphone sensor data and survey features included in the linear regression model to predict Nomophobia Questionnaire total scores among 27 participants with depressive symptoms over the 8-week study period, reaching an *R^2^* value of 0.779.

Rank	Feature	Sensor or questionnaire	Attribute coefficient
1	Count of social media app use	App	−17.55
2	Sleep disturbance	QIDS^a^	−4.40
3	Count of sent messages to the most frequent contact	Message	−1.5
4	The total traveled distance	Location	−1.32
5	SD of unlock episode duration	Screen	0.81
6	The maximum period staying at significant locations	Location	0.03

^a^QIDS: Quick Inventory of Depressive Symptomatology.

**Table 8:** Overview of smartphone sensor features included in the linear regression model to predict Nomophobia Questionnaire total scores among 27 participants with depressive symptoms over the 8-week study period, reaching an *R^2^* value of 0.732.

Rank	Feature	Sensor or questionnaire	Attribute coefficient
1	Count of social media app use	App	−20.59
2	Count of sent messages to the most frequent contact	Message	−2.23
3	SD of unlock episode duration	Screen	0.81
4	The maximum period of staying at significant locations	Location	0.02
5	The area covered by a participant	Location	–0.0005
6	The total traveled distance	Location	–0.00000218
7	The average amount of items scrolled	Touch	0.00000158

Both models’ performance was mostly related to the frequency of social media app use. The count of sent messages to the most frequent contact, the duration of staying at clusters, and the SD of the duration of screen unlock episodes were also important features revealing smartphone use and location information in both models.

## Discussion

### Themes

The results from our exploratory study are suggestive of several themes concerning nomophobia and its associations with both psychopathology and smartphone use.

#### Depression and Negative or Positive Affect

Given that the SMARTSENSE-D study focused on a cohort of young people diagnosed with clinical depression and varying degrees of symptom severity, the data provided a good opportunity to explore associations between nomophobia and depression. The 2 psychometric scales concerning depression that were used in the study, the DASS-21 and QIDS, both showed significant associations with nomophobia. The DASS-21 depression scale and 2 QIDS factors positively correlated with total nomophobia scores.

Our EMA results across the study indicate that the more positive affect one has in general, the lower their nomophobia. The most salient of these results concern happiness and relaxation, for which the EMA results negatively correlated with total nomophobia scores.

These findings regarding associations among depression, affect, and nomophobia are in line with previous findings [61-67]. Given the co-occurrence of depression and nomophobia, there is a possibility that depressive symptoms and negative affect are influenced by nomophobia, or vice versa. The connection and causality among depression, negative feelings, and nomophobia need to be further validated through additional research.

#### Location

Our results suggest that participants who spent more time at home or at their otherwise most visited location had greater nomophobia. Relatedly, one’s maximum duration at a significant location also positively correlated with nomophobia. This implies that the more an individual is confined to their home or the more concentrated their geolocation to a particular location, the greater their sense of dependency on their smartphone.

Our results also showed a positive correlation between nomophobia and the SD of all the durations of time that a participant spent at their significant locations for the day. This is a result that is perhaps less straightforward to interpret; we can say that, if one has a lower SD, then there is a more evenly spread diversity in the time they spend at their various significant locations. This contrasts with a higher SD, which implies a concentration in fewer places. In this case, the SD result is in line with the other geolocation results discussed previously.

The fact that nomophobia was associated both with geolocation and depressive symptoms is in line with previous research that has demonstrated a significant link between movement patterns and depression [56,68-70]. Thus, a triangle emerges that connects geolocation, depression, and nomophobia. Ultimately, limited or confined geolocation activity is associated with nomophobia and can be an indicator of depression.

#### Nomophobia-Privacy Trade-Off

A trend of positive correlations emerged between nomophobia and comfort with being tracked via a smartphone sensing app and sharing data. The NMP-Q “giving up convenience” dimension positively correlated with comfort levels with using the AWARE-Light sensing app and activating sensors within it.

Thus, a trade-off seems to emerge whereby individuals with nomophobic tendencies, particularly their concern of giving up the convenience afforded by smartphones, are less concerned about being smartphone sensed. Alternatively, one could perhaps frame this as being about privacy concerns outweighing certain smartphone benefits and that those more concerned about privacy are less inclined to develop nomophobia.

At any rate, these findings relate to those of previous studies that have shown how privacy concerns can influence smartphone use behaviors as a dependent indicator [71,72]. Our results from the self-reported evaluations of both participants’ nomophobia levels and attitudes toward privacy further support this correlation between smartphone use and privacy concerns.

#### Smartphone Use

Previous studies [27] have indicated a positive correlation between nomophobia and problematic smartphone use, including screen status (eg, screen locks and unlocks and turning the screen on or off). While none of our formal results involving screen sensor features were significant, we did observe using a scatter plot that, when limited to participants in the mild and severe NMP-Q categories (moderate category excluded), there was a noticeable positive correlation between the number of phone unlocks and NMP-Q scores. However, further work is required to formally strengthen the case that nomophobia is associated with greater screen use, such as unlock instances.

### Predictive Models

The predictive results shown by our linear regression models indicate the feasibility of predicting the NMP-Q score based on passive human behavioral features. The implications of these results can be valuable for the understanding, detection, and control of nomophobia.

The most important feature in both models, the count of social media app use, showed a negative relationship with the predicted ground-truth NMP-Q score, indicating that engagement with social media apps co-occurs with a lower level of nomophobia. Meanwhile, the count of sent messages to the most frequent contact and the total traveled distance demonstrated different levels of negative association with the NMP-Q. These findings should be highlighted for further exploration regarding the role of social media apps in managing the relationship between users and smartphones.

### Limitations

Our primary goal was to understand the relationship between the features and the NMP-Q, demonstrating the feasibility of predicting NMP-Q scores based on smartphone sensor data and psychometrics or even using only passively collected data. However, we acknowledge that more complex models such as Extreme Gradient Boosting or support vector machines might be valuable in future research with more data to explore potential nonlinear relationships within the data. These advanced models could offer deeper insights and improve prediction accuracy by capturing intricate patterns that simpler linear models might miss.

This study was exploratory and aimed to gain preliminary insights into associations between smartphone-inferred behavioral features and nomophobia (NMP-Q score), as well as the potential of machine learning models to predict NMP-Q scores from such features. The primary intent was not to provide a model ready for practical application at this stage but to demonstrate the feasibility of model use in future stages with more extensive data over a longer time frame. Predictive models for NMP-Q score could assist researchers, patients with depression, and mental health professionals in understanding the impact of smartphone use and daily behaviors on mental health from a technological perspective.

Our inferential statistical results provide a starting point for more dedicated studies with larger samples sizes to strengthen the claims suggested by our results. The use of a 20% false discovery rate may have introduced potential false positives, especially in a small cohort of participants. This threshold aimed to balance false positives and false negatives. However, to achieve more robust and precise conclusions, the findings need further confirmation with larger sample sizes. The larger datasets would also incorporate more advanced machine learning techniques and be less prone to issues such as overfitting. With more data and refined models, clinicians could better understand depression and its relationship with smartphone use, aiding those with depressive symptoms. This could offer valuable insights into how these digital interactions influence their lives and aid in better mental health management.

In addition to the small sample size, another limitation was the imperfection of our data collection given technical issues encountered, issues with AWARE-Light in particular, and the general difficulty of getting smartphone sensing data collection to work across various sensors and various participant phones. Thus, we had to implement a data quality filtering procedure to ensure that we only used a phone’s data for a given sensor if those data met certain threshold requirements. Consequently, not all participants were included for every sensor.

### Conclusions

Our study explored associations among nomophobia, clinical measures, EMAs, smartphone sensor data, and smartphone use questionnaires. We discovered that greater nomophobia is linked to more depressive symptoms, limited geolocation movement and (domestic) stationariness, comfort with being smartphone sensed, and negative emotions. In doing so, we demonstrated that smartphone sensing can effectively quantify user behaviors and perceptions underlying nomophobia and the phenomena that its measures are designed to capture.

Nomophobia is a nascent notion that merits further investigation. Beyond conceptualizations and measurement scales, use of the technology in question (ie, smartphones) to provide objective quantification of nomophobic-related use and behavioral phenomena requires further exploration through larger and more in-depth purpose-built studies. It is hoped that these initial exploratory findings will contribute to paving the way for such future work.

## Appendix

Multimedia Appendix 1 Questions presented in the debriefing questionnaire administered at the end of the 8-week study. Items 1 to 20 are provided in scales from 1 to 7 indicating the level of agreement. Items 21 to 28 are provided in scales from 1 to 7 indicating the comfort level of the participants with the sensor being activated on their smartphones. Items 29 and 30 are provided as Yes or No options.

Multimedia Appendix 2 Daily ecological momentary assessment items for 41 participants with major depressive disorder. The 21-question survey, administered at noon and 8 PM, used 7-point Likert-scale items (questions 1-14 and 18), binary (yes or no) items (questions 15 and 19), and open-text responses (questions 16, 17, 20, and 21).

Multimedia Appendix 3 Comprehensive correlation analysis results between Nomophobia Questionnaire total scores and various study variables, including psychometric measures assessed at the end of the study and smartphone sensing features recorded throughout the study. The analysis includes 41 participants for the psychometric data and 27 participants for variables involving smartphone sensor features, including both significant and nonsignificant associations.

Multimedia Appendix 4 Preselected features used in predictive models to estimate Nomophobia Questionnaire total scores for 27 participants with major depressive disorder. These features include psychometric measures collected at the end of the 8-week study and smartphone-derived variables recorded throughout the study.

## Abbreviations

DASS-21: Depression, Anxiety, and Stress Scale–21
EMA: ecological momentary assessment
HDBSCAN: Hierarchical Density-Based Spatial Clustering of Applications With Noise
MAE: mean absolute error
MAPE: mean absolute percentage error
MDD: major depressive disorder
NMP-Q: Nomophobia Questionnaire
PSWQ: Penn State Worry Questionnaire
QIDS: Quick Inventory of Depressive Symptomatology
RMSE: root mean square error
RRS: Rumination Response Scale
UCLA: University of California, Los Angeles

## Data Availability

The datasets generated or analyzed during this study are not publicly available because not all participants in this study provided consent for their data to be openly shared. For the remaining participants, the data are available from the corresponding author on reasonable request.
