# Supplementary material for: Nomophobia, Psychopathology, and Smartphone-Inferred Behaviors in Youth With Depression: Longitudinal Study
Source: JMIR Form Res. 2025 Feb 19;9:e57512. doi: 10.2196/57512 (PMC11888105; doi:10.2196/57512)
Supplement: Multimedia Appendix 1 [file formative_v9i1e57512_app1.docx]

| Item | Description |
| --- | --- |
| 1 | It was easy to learn to use the app. |
| 2 | I thought the app was easy to use. |
| 3 | I understand how the app works. |
| 4 | I felt comfortable using the app. |
| 5 | The app did not interfere with the operation of my smartphone. |
| 6 | I generally kept my smartphone with me. |
| 7 | Using the app made me more conscious of the way I use my smartphone. |
| 8 | I was concerned about my privacy using the app. |
| 9 | I was comfortable with having my information collected by the app. |
| 10 | Using the app made me think about my mental health condition more. |
| 11 | Using the app made me upset. |
| 12 | If future studies could create a summary of associations between my smartphone usage and mood changes, I would like to see it. |
| 13 | If future studies could create a summary of associations between my smartphone usage and mood changes, I would be comfortable to share it with my clinician. |
| 14 | If future studies could give me daily feedback about my behavior, I would want to receive it. |
| 15 | If future studies could monitor the risk of experiencing another depressive episode via passive sensing, I would like to take part. |
| 16 | The frequency of the phone questionnaire was acceptable. |
| 17 | The additional drainage of my phone’s battery by the AWARE app was acceptable. |
| 18 | Wearing the actigraphy device for 8 weeks was comfortable. |
| 19 | Duration of the phone questionnaire was acceptable. |
| 20 | Delivery times of the phone questionnaires were acceptable. |
| 21 | Accelerometer - Measures phone movement. |
| 22 | Applications - Captures which apps are used and when. |
| 23 | Communication - Captures number of incoming/outgoing/unanswered calls and SMSs. |
| 24 | Location - Captures location. |
| 25 | Light - Compares amount of light surrounding the phone. |
| 26 | Keyboard - Records speed of typing without content of typing being recorded. |
| 27 | Network - Senses the nearby networks (e.g. Wi-Fi, Bluetooth, hotspots). |
| 28 | Screen usage - Captures when you lock or unlock your screen. |
| 29 | During the past 8 weeks, did you turn off any of the sensors through the settings in AWARE app? |
| 30 | Did you make any changes to the way you normally use your phone, knowing that your phone usage was monitored? |

Table A1.: Questions presented in the debriefing questionnaire administered at the end of the 8-week study. Items 1-20 are provided scales from 1 to 7 indicating the level of agreement. Items 21-28 are provided scales from 1 to 7 indicating the comfort level participants felt with the sensor being activated on their smartphones. Item 29-30 are provided options Yes or No.
