# Supplementary material for: Nomophobia, Psychopathology, and Smartphone-Inferred Behaviors in Youth With Depression: Longitudinal Study
Source: JMIR Form Res. 2025 Feb 19;9:e57512. doi: 10.2196/57512 (PMC11888105; doi:10.2196/57512)
Supplement: Multimedia Appendix 2 [file formative_v9i1e57512_app2.docx]

| Item | Question | EMA at 12:00 pm | EMA at 8:00 pm |
| --- | --- | --- | --- |
| 1 | How cheerful did you feel? | ✓ | ✓ |
| 2 | How happy did you feel? | ✓ | ✓ |
| 3 | How excited did you feel? | ✓ | ✓ |
| 4 | How relaxed did you feel? | ✓ | ✓ |
| 5 | How sad did you feel? | ✓ | ✓ |
| 6 | How guilty did you feel? | ✓ | ✓ |
| 7 | How angry did you feel? | ✓ | ✓ |
| 8 | How nervous did you feel? | ✓ | ✓ |
| 9 | How stressed did you feel? |  | ✓ |
| 10 | How anxious did you feel? |  | ✓ |
| 11 | How worried did you feel? |  | ✓ |
| 12 | How much did you think about problems you have? |  | ✓ |
| 13 | How lonely did you feel? |  | ✓ |
| 14 | How much did you interact with other people? |  | ✓ |
| 15 | Did you use substances (including alcohol) TODAY? |  | ✓ |
| 16 | If yes to question 15, which substance(s) did you use? |  | ✓ |
| 17 | If there were any significant events today that may have impacted your mood, behavior or phone usage, you can explain below. |  | ✓ |
| 18 | How well did you sleep last night? | ✓ |  |
| 19 | Did you use substances (including alcohol) LAST NIGHT after you filled in the survey? | ✓ |  |
| 20 | If yes to question 19, which substance(s) did you use? | ✓ |  |
| 21 | If there were any significant events last night that may have impacted your mood, behavior or phone usage, you can explain below. | ✓ |  |

Table A2.: Questions presented in the Ecological Momentary Assessments (EMA) administered daily to 41 participants with major depressive disorder. Each set of EMA sessions consisted of 21 questions designed to capture participants’ experiences at 12:00 pm and 8:00 pm per day. Questions 1-8 assessed participants’ experiences since midday and up until answering the questionnaire, while questions 9-17 captured their overall experience of the day. The EMA delivered at 12:00 pm captured participants’ overall experience from waking up until answering the questionnaire. Responses to questions 1-14 and 18 were given on a Likert scale with values ranging from 1 to 7, while questions 15 and 19 were binary (Yes/No) questions. Questions 16, 17, 20 and 21 were open-ended textual questions.
