# Supplementary material for: Nomophobia, Psychopathology, and Smartphone-Inferred Behaviors in Youth With Depression: Longitudinal Study
Source: JMIR Form Res. 2025 Feb 19;9:e57512. doi: 10.2196/57512 (PMC11888105; doi:10.2196/57512)
Supplement: Multimedia Appendix 3 [file formative_v9i1e57512_app3.docx]

| Category | Combination | | P value | Coefficient |
| --- | --- | --- | --- | --- |
|  | Feature of the category | NMP-Q scale |  |  |
| Location sensor | average speed  the minimum time spent at significant locations  the number of significant places  total travelled distance  **the maximum time spent at significant locations**  **the standard deviation of time spent at significant locations**  **time spent at home**  **time spent at the most significant location** | total  total  total  total  **total**  **total**  **total**  **total** | 0.976  0.072  0.456  0.224  **0.023**  **0.039**  **0.023**  **0.025** | -0.005  0.284  0.120  0.194  **0.354**  **0.323**  **0.353**  **0.350** |
| Screen sensor | total duration of all unlock episodes  the longest duration of any unlock episode  the number of all unlock episodes | total  total  total | 0.547  0.783  0.668 | 0.097  0.044  0.069 |
| EMA | the average between the morning and evening scores for cheer  the average between the morning and evening scores for excitement  the average between the morning and evening scores for sadness the average between the morning and evening scores for guilt  the average between the morning and evening scores for anger  the average between the morning and evening scores for nervousness | total  total  total  total  total  total | 0.072  0.148  0.695  0.997  0.795  0.747 | -0.284  -0.230  0.063  0.5e-4  0.042  0.052 |
| Debriefing questions | participant comfort with using AWARE-Light | subfactor: not being able to communicate (belongings and connectedness connectedness) | 0.969 | -0.006 |
|  | participant comfort with sensor activation | subfactor: not being able to communicate (belongings and connectedness) | 0.796 | 0.042 |
|  | participant comfort with using AWARE-Light | subfactor: losing connectedness | 0.101 | 0.259 |
|  | participant comfort with sensor activation | subfactor: losing connectedness | 0.064 | 0.292 |
|  | participant comfort with using AWARE-Light | subfactor: not being able to access information | 0.155 | 0.226 |
|  | participant comfort with sensor activation | subfactor: not being able to access information | 0.276 | 0.174 |
|  | participant comfort with using AWARE-Light | total | 0.125 | 0.243 |
|  | participant comfort with sensor activation | total | 0.114 | 0.250 |
|  | **participant comfort with using AWARE-Light** | **subfactor: giving up convenience** | **0.017** | **0.372** |
|  | **participant comfort with sensor activation** | **subfactor: giving up convenience** | **0.010** | **0.395** |
| DASS | total  subfactor: anxiety  subfactor: stress  **subfactor: depression** | total  total  total  **total** | 0.293  0.924  0.686  **0.016** | 0.168  0.015  0.065  **0.375** |
| QIDS | total  subfactor: sleep disturbance  **subfactor: mood**  subfactor: concentration  subfactor: self-criticism  subfactor: suicidal ideation  subfactor: interest  **subfactor: energy/fatigue**  subfactor: psychomotor agitation/retardation | total  total  **total**  total  total  total  total  **total**  total | 0.090  0.557  **0.046**  0.088  0.287  0.255  0.344  **0.018**  0.980 | 0.268  -0.095  **0.313**  0.270  0.170  0.182  0.152  **0.367**  0.004 |
| RRS | total | total | 0.355 | 0.148 |
| PSWQ | total | total | 0.096 | 0.264 |
| UCLA | total | total | 0.615 | -0.081 |

Table A3.: Comprehensive correlation analysis results between Nomophobia Questionnaire (NMP-Q) total scores and various study variables, including psychometric measures assessed at the end of the study and smartphone sensing features recorded throughout the study. The analysis includes 41 participants for psychometric data and 27 participants for variables involving smartphone sensor features, including both significant and non-significant associations.
