# Supplementary material for: Nomophobia, Psychopathology, and Smartphone-Inferred Behaviors in Youth With Depression: Longitudinal Study
Source: JMIR Form Res. 2025 Feb 19;9:e57512. doi: 10.2196/57512 (PMC11888105; doi:10.2196/57512)
Supplement: Multimedia Appendix 4 [file formative_v9i1e57512_app4.docx]

| Category | Feature | Applied in best models |
| --- | --- | --- |
| DASS | total  subfactor: depression  subfactor: anxiety subfactor: stress |  |
| QIDS | total  subfactor: sleep disturbance subfactor: mood  subfactor: appetite/weight subfactor: concentration subfactor: self-criticism  subfactor: suicidal ideation subfactor: interest  subfactor: energy/fatigue  subfactor: psychomotor agitation/retardation | ✓ |
| UCLA | total |  |
| PSWQ | total |  |
| RRS | total |  |
| Touch sensor | the average number of items scrolled | ✓ |
| Location sensor | the number of movements between any two location clusters  the maximum time spent at significant locations  speed variance  average speed  location variance  the standard deviation of time spent in significant locations  time spent at home  location entropy  average time spent at the most significant location  covered area  the number of significant places  the average period spent at significant locations  the total travelled distance | ✓ |
|  |  | ✓ |
|  |  | ✓ |
| Message | the count of received messages to the most frequent contact  the count of received messages  the number of distinct contacts with received messages  the count of sent messages to the most frequent contact  the count of sent messages  the number of distinct contacts with sent messages | ✓ |
| Screen sensor | the count of unlock episodes  the sum duration of unlock episode  the maximum duration of unlock episode the minimum duration of unlock episode  the average duration of unlock episode  the standard deviation of unlock episode duration | ✓ |
| Application sensor | the count of social app usage  the count of communication app usage the count of entertainment app usage the count of music/audio app usage | ✓ |

Table A4.: Pre-selected features used in predictive models to estimate Nomophobia Questionnaire (NMP-Q) total scores for 27 participants with major depressive disorder. These features include psychometric measures collected at the end of the 8-week study and smartphone-derived variables recorded throughout the study.
